# Management of Ureterolithiasis in a Patient with Crossed Unfused Renal Ectopia

**DOI:** 10.1155/2016/1847213

**Published:** 2016-06-14

**Authors:** Koichi Kodama, Yasukazu Takase, Hiroki Tatsu

**Affiliations:** ^1^Department of Urology, Toyama City Hospital, Toyama 939-8511, Japan; ^2^Department of Radiology, Toyama City Hospital, Toyama 939-8511, Japan

## Abstract

Crossed renal ectopia is a rare congenital anomaly in which both kidneys are situated on one side and fused together in 85%–90% of cases. The management of urinary calculi in patients with crossed renal ectopia continues to pose challenges to urologists because the aberrant anatomy may make access and clearance of the calculi more difficult to accomplish. Here, we report a case of inferior crossed renal ectopia, without fusion, and a ureteral stone in which the patient was treated successfully by extracorporeal shock wave lithotripsy.

## 1. Introduction

Crossed renal ectopia is a rare congenital anomaly in which both kidneys are situated on one side and fused together in 85%–90% of cases. The management of urinary calculi in patients with crossed renal ectopia continues to pose challenges to urologists because the aberrant anatomy may make access and clearance of the calculi more difficult to accomplish. The treatment options for these calculi in such situations include extracorporeal shock wave lithotripsy (ESWL), percutaneous lithotomy, and laparoscopy [1–3]. There have been reports on the effectiveness and safety of ureteroscopy with laser lithotripsy for the management of renal calculi [4]. Here, we report a case of inferior crossed renal ectopia without fusion and ureterolithiasis in which the patient was treated successfully by ESWL.

## 2. Case Presentation

**Figure 1:**
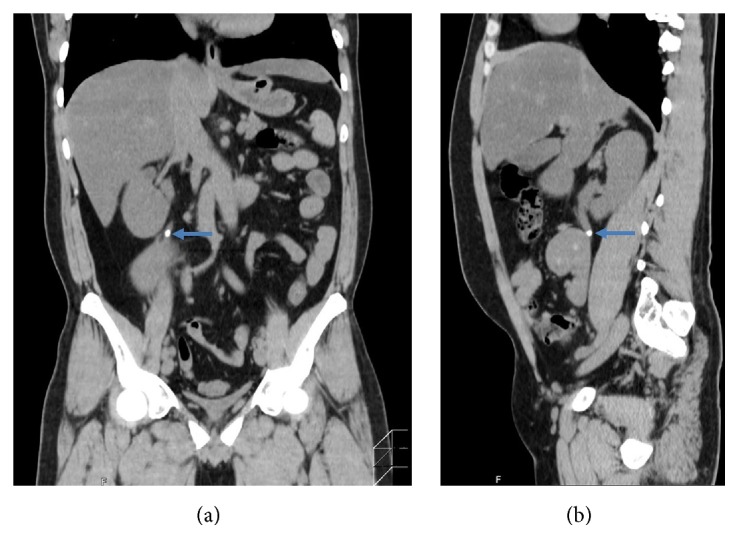
Coronal (a) and sagittal (b) sections of unenhanced computed tomography of the abdomen show left-to-right crossed unfused renal ectopia and a 5 mm stone (arrows) in the proximal ureter of the kidney located cranially. A left inferior vena cava is also seen.

**Figure 2:**
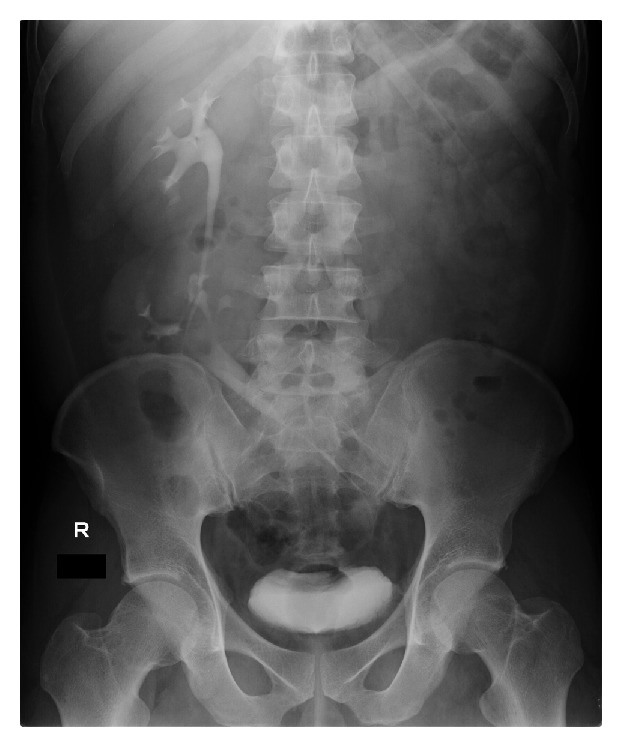
One month after the extracorporeal shock wave lithotripsy, intravenous urography confirms the absence of any residual stone. The ureter of the ectopic kidney is seen to cross the midline and enter into the urinary bladder at the normal position.

A 32-year-old man presented with right flank pain. He had an unremarkable medical history. The patient described a stabbing pain on the right side of his back. On detailed physical examination, no congenital anomaly was found. Urinalysis revealed microscopic hematuria. Ultrasonography showed that both kidneys were on the right side of the abdomen. Fatty liver and slight dilatation of the pelvicalyceal system of the kidney located cranially, which was caused by a ureteral stone, were also found. Unenhanced computerized tomography (CT) of the abdomen showed left-to-right crossed renal ectopia and a 5 mm stone in the proximal ureter of the kidney located cranially (Figure 1). The crossed ectopic kidney was located inferior to the nonectopic kidney without fusion. The ureter of the ectopic kidney was placed transversely and anteriorly to the promontory. In addition, a left inferior vena cava (IVC) with hemiazygos continuation was also found. However, no anatomical peculiarities were detected in CT that could have been a cause for the development of stone. ESWL under continuous ultrasound imaging was performed as an outpatient procedure. One month after the ESWL session, intravenous urography confirmed the absence of any residual stones (Figure 2). Laboratory data suggested that the patient had no risk factors for urinary calculi. Stone composition could not be identified because the small stone fragments were not caught.

## 3. Discussion

Crossed renal ectopia is a rare congenital anomaly in which both kidneys are situated on one side. The ureter of the crossed ectopic kidney recrosses the midline and enters the bladder on the opposite but normal side. This is thought to result from the abnormal development and migration of the ureteric bud and metanephric blastema during the fourth week to eighth week of gestation. In general, crossed renal ectopia is found incidentally when patients are investigated for other abdominal pathologies, and it can have various presentations. In some cases, it may be associated with urinary calculi, recurrent infections, and hydronephrosis. There is a male predominance of 3 : 2, and left-to-right crossover occurs more frequently than right-to-left crossover [5].

Crossed unfused renal ectopia is a rare type of renal fusion anomaly. McDonald and McClellan classified crossed renal ectopia into (i) crossed ectopia with fusion, (ii) crossed ectopia without fusion, (iii) unilateral crossed ectopia (associated with unilateral renal agenesis), and (iv) bilateral crossed ectopia without fusion (both ureters cross the midline) [6]. The kidneys are fused together in 85%–90% of cases [7]. The incidence of the unfused variety has been reported to be 1 in 75,000 autopsies [8].

It is important to note that renal ectopia is frequently associated with congenital anomalies of other organ systems. Genetic factors may also play a role [9]. In the present case, the left IVC ascended and joined the renal vein of the ectopic kidney. The IVC crossed to the right side at approximately the level of the renal artery of the nonectopic kidney, crossing in front of the aorta. The demonstration of accompanying anomalies as well as a congenital renal anomaly would also be important for operative planning before renal and aortic surgery [10].

The management of urinary calculi in patients with congenital renal anomalies, such as crossed renal ectopia, continues to pose challenges to urologists because the aberrant anatomy may make access and clearance of the calculi more difficult to accomplish. ESWL remains the first choice of treatment for these calculi because it is the least invasive treatment modality. However, multiple sessions are often required before patients become free of calculi [1]. More recently, there have been reports on the effectiveness and safety of flexible ureteroscopy with laser lithotripsy for the management of urinary calculi [4]. In the present case, the ureteral stone was not on the side of the renal ectopia. If the stone is on the side of the crossed renal ectopia, it needs good pretreatment planning, including the need of flexible ureteroscopy. In these circumstances, one may even consider stone push-up by flexible ureteroscopy combined with minipercutaneous nephrolithotomy. The choice of treatment for urinary calculi in cases of crossed renal ectopia should be made depending on the size and position of the urinary calculi and the patient's anatomy.
